# Dataset of the absorption, emission and excitation spectra and fluorescence intensity graphs of fluorescent cyanine dyes for the quantification of low amounts of dsDNA

**DOI:** 10.1016/j.dib.2016.11.090

**Published:** 2016-11-28

**Authors:** Brigitte Bruijns, Roald Tiggelaar, Han Gardeniers

**Affiliations:** a Mesoscale Chemical Systems, MESA^+^ Institute for Nanotechnology, University of Twente, Enschede, The Netherlands; b Life Science, Life Science, Engineering & Design, Saxion University of Applied Sciences, Enschede, The Netherlands

## Abstract

This article describes data related to a research article entitled “Fluorescent cyanine dyes for the quantification of low amounts of dsDNA” (B. Bruijns, R. Tiggelaar, J. Gardeniers, 2016) [1]. Six cyanine dsDNA dyes - EvaGreen, SYBR Green, PicoGreen, AccuClear, AccuBlue NextGen and YOYO-1 – are investigated and in this article the absorption spectra, as well as excitation and emission spectra, for all six researched cyanine dyes are given, all recorded under exactly identical experimental conditions. The intensity graphs, with the relative fluorescence in the presence of low amounts of dsDNA, are also provided.

**Specifications Table**

**Table:** 

Subject area	*Chemistry, Biology*
More specific subject area	*Fluorescent cyanine dyes for the quantification of low amounts of dsDNA*
Type of data	*Table, figure*
How data was acquired	*Microplate reader and cuvette measurements*
Data format	*Processed*
Experimental factors	*Type of dye, amount of DNA*
Experimental features	*Spectra (absorption, excitation and emission) and fluorescence intensity*
Data source location	*Enschede, The Netherlands*
Data accessibility	*Data is given in this article*

**Value of the data**

• The optimal wavelengths of absorption, emission and excitation of six cyanine dyes - EvaGreen, SYBR Green, PicoGreen, AccuClear, AccuBlue NextGen and YOYO-1 – are determined for identical experimental settings and spectral data is given in this article.
• The fluorescence intensities of these cyanine dyes with low amounts of dsDNA (pg–ng range) are recorded and if present the linear ranges are reported in the datasets in this article.
• For AccuClear and AccuBlue NextGen this is, as far as known by the authors, the first set of data in an academic journal.

## 1 Data

Cyanine dyes can be used to quantify the amount of dsDNA within a sample. The linearity of fluorescence, as function of DNA amount of six dyes, is obtained by measuring the fluorescence intensity at the optimal excitation and emission maxima.

In Appendix A of Supplementary material a detailed overview is given about the characteristics and spectral behaviour of the researched dyes, such as the absorption, excitation and emission wavelengths at which maxima occur. The wavelengths at which these maxima occur as available in literature are listed in Table 1.1 and the measured dataset of the absorption, emission, excitation and fluorescence intensity graphs are given in Fig. 2.1, Fig. 2.2, Fig. 2.3, Fig. 2.4, Fig. 2.5, Fig. 2.6, Fig. 2.7, Fig. 2.8, Fig. 2.9, Fig. 2.10, Fig. 2.11, Fig. 2.12, Fig. 2.13, Fig. 2.14, Fig. 2.15, Fig. 2.16, Fig. 2.17, Fig. 2.18, Fig. 2.19, Fig. 2.20, Fig. 2.21, Fig. 2.22, Fig. 2.23, Fig. 2.24, Fig. 2.25, Fig. 2.26^1^ (and listed in Table 3 in [1]).

## 2 Experimental design, materials and methods

The materials and methods used to obtain the dataset of the absorption, excitation, emission and fluorescence intensity graphs are given in [1].

## Figures and Tables

**Fig. 2.1:**
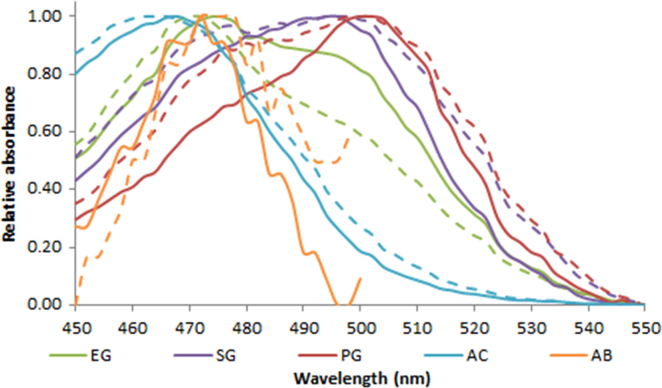
Absorption spectra of 1.0X EvaGreen (green lines), SYBR Green (purple lines), PicoGreen (red lines), AccuClear (aqua lines) and AccuBlue NextGen (orange lines) free dye (dash lines) and in the presence of 10 ng (AC, AB) or 100 ng (EG, SG and PG) salmon dsDNA (solid lines). Spectra were recorded with a M200 PRO microplate reader (Tecan).

**Fig. 2.2:**
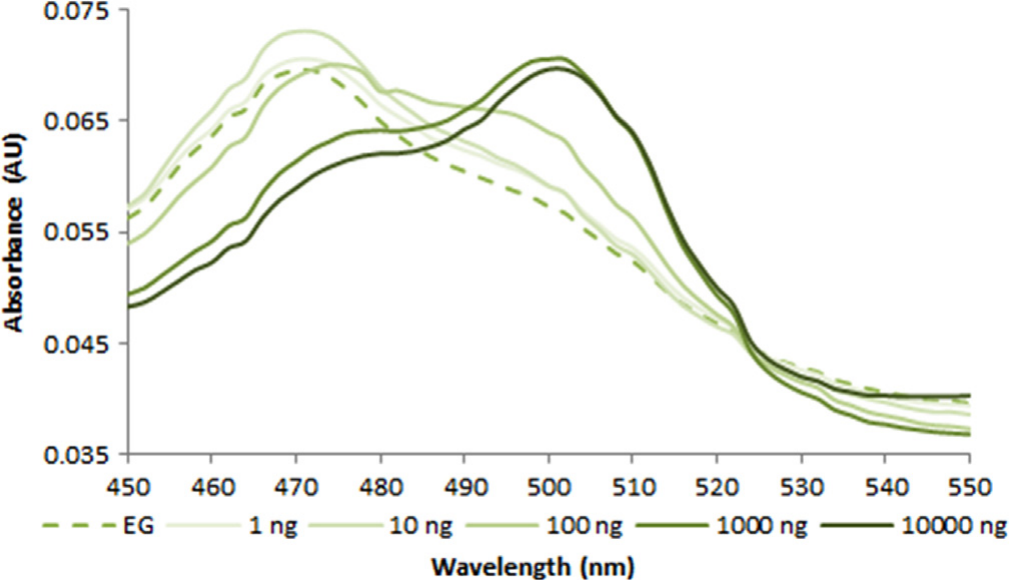
Absorption spectra of 1.0X EvaGreen free dye (dash line) and in the presence of various amounts of dsDNA (solid lines). Spectra were recorded with a M200 PRO microplate reader (Tecan).

**Fig. 2.3:**
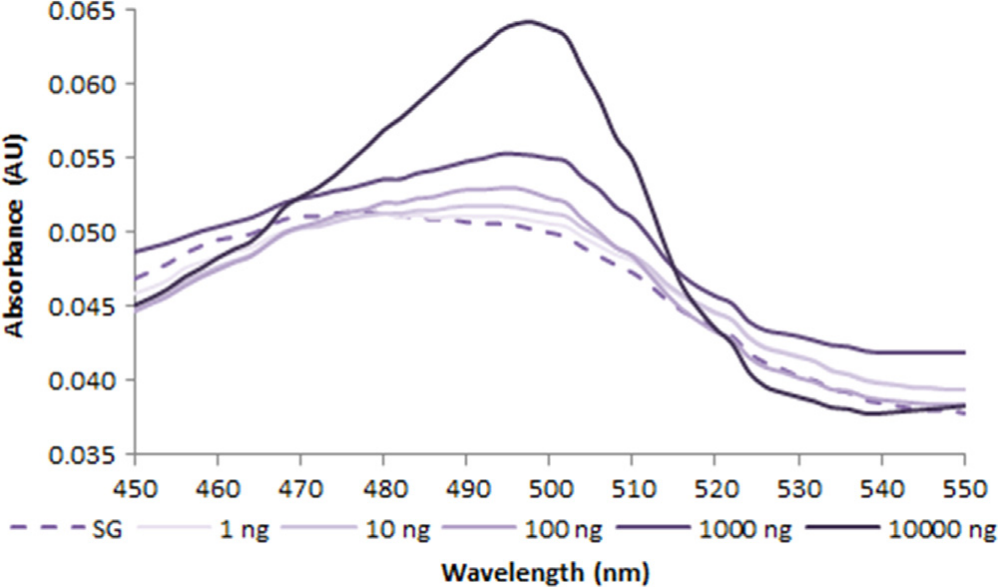
Absorption spectra of 1.0X SYBR Green free dye (dash line) and in the presence of various amounts of dsDNA (solid lines). Spectra were recorded with a M200 PRO microplate reader (Tecan).

**Fig. 2.4:**
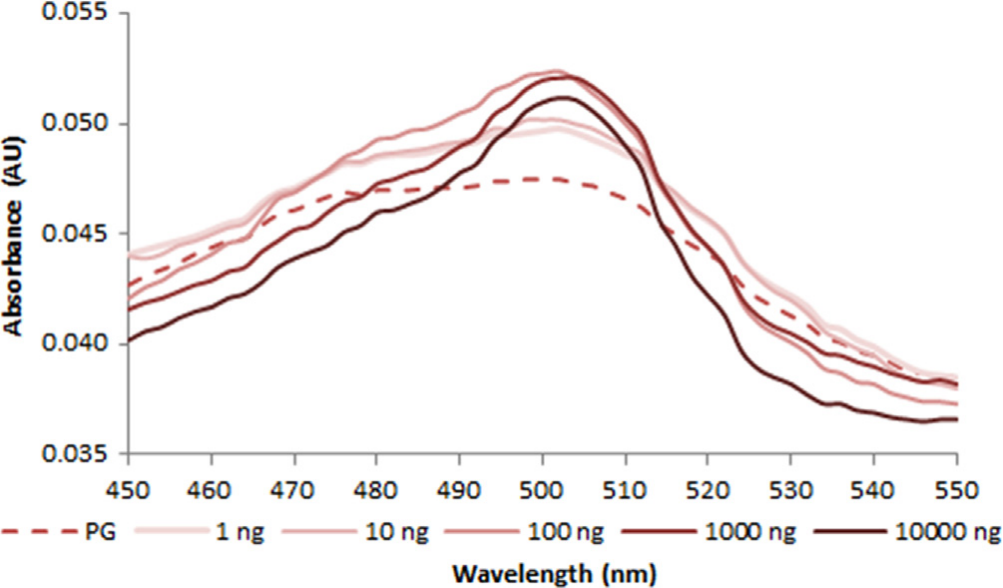
Absorption spectra of 1.0X PicoGreen free dye (dash line) and in the presence of various amounts of dsDNA (solid lines). Spectra were recorded with a M200 PRO microplate reader (Tecan).

**Fig. 2.5:**
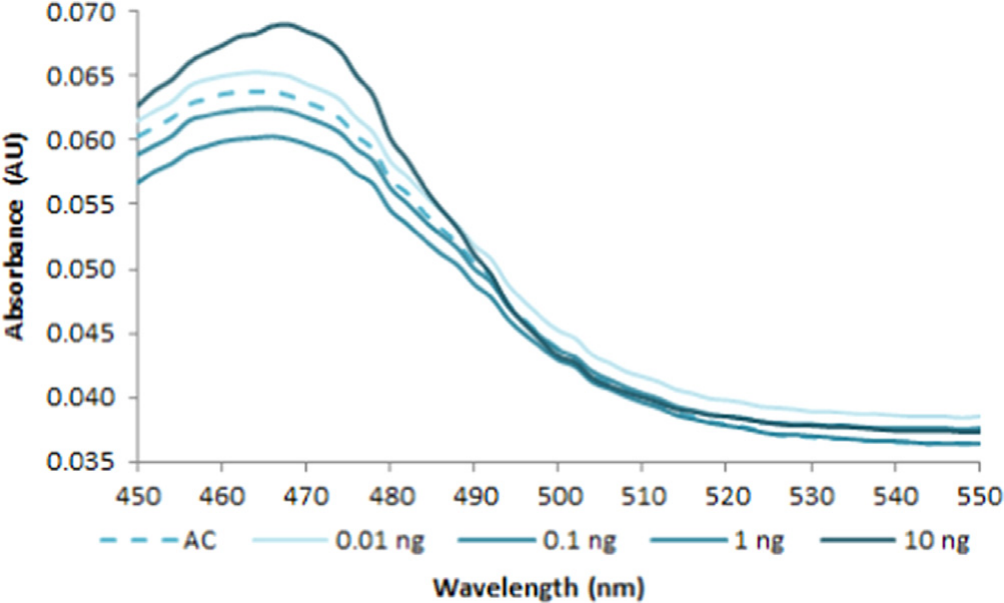
Absorption spectra of 1.0X AccuClear free dye (dash line) and in the presence of various amounts of dsDNA (solid lines). Spectra were recorded with a M200 PRO microplate reader (Tecan).

**Fig. 2.6:**
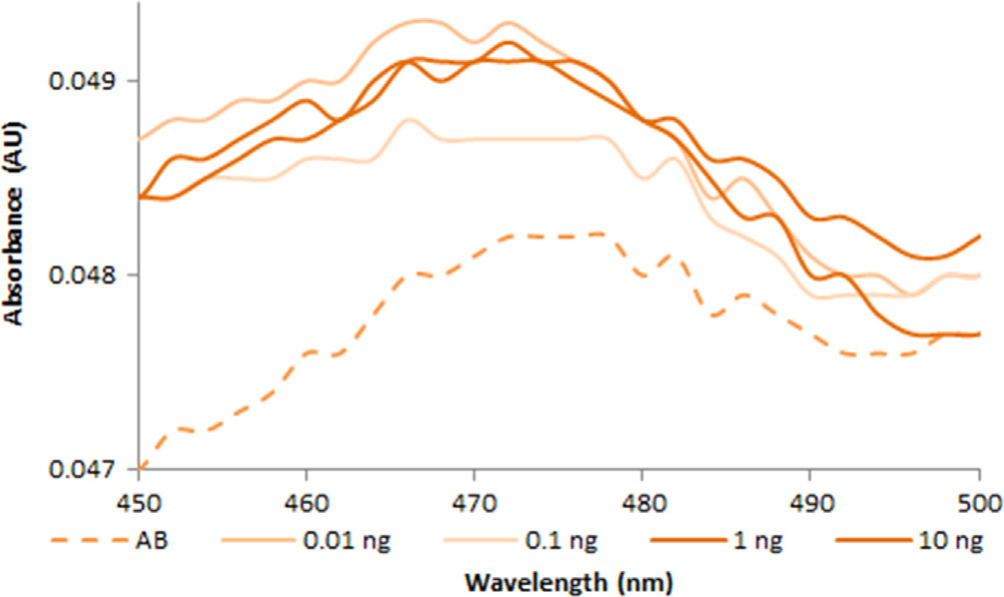
Absorption spectra of 1.0X AccuBlue NextGen free dye (dash line) and in the presence of various amounts of dsDNA (solid lines). Spectra were recorded with a M200 PRO microplate reader (Tecan).

**Fig. 2.7:**
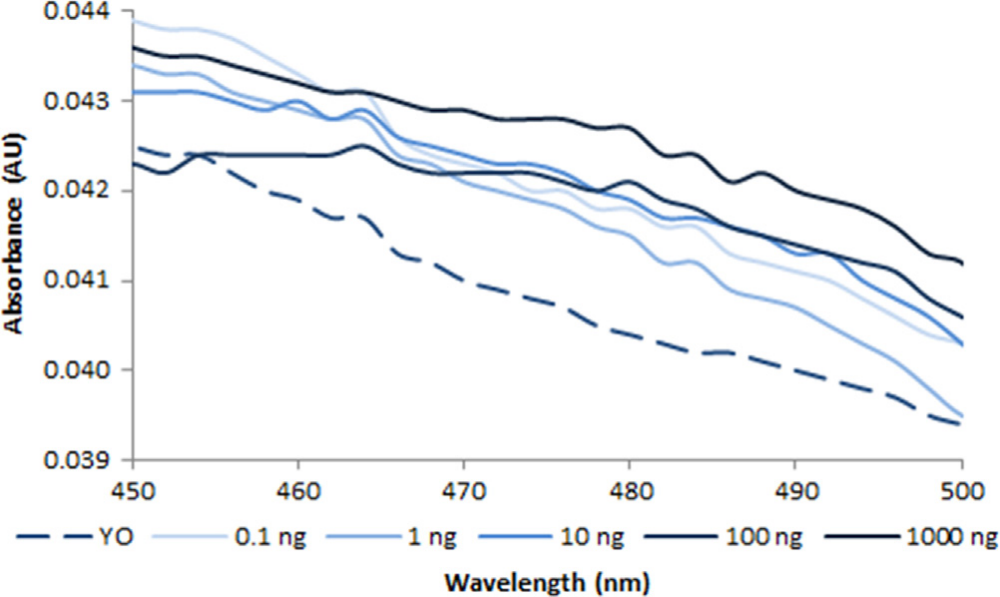
Absorption spectra of 100 nM YOYO-1 free dye (dash line) and in the presence of various amounts of dsDNA (solid lines). Spectra were recorded with a M200 PRO microplate reader (Tecan).

**Fig. 2.8:**
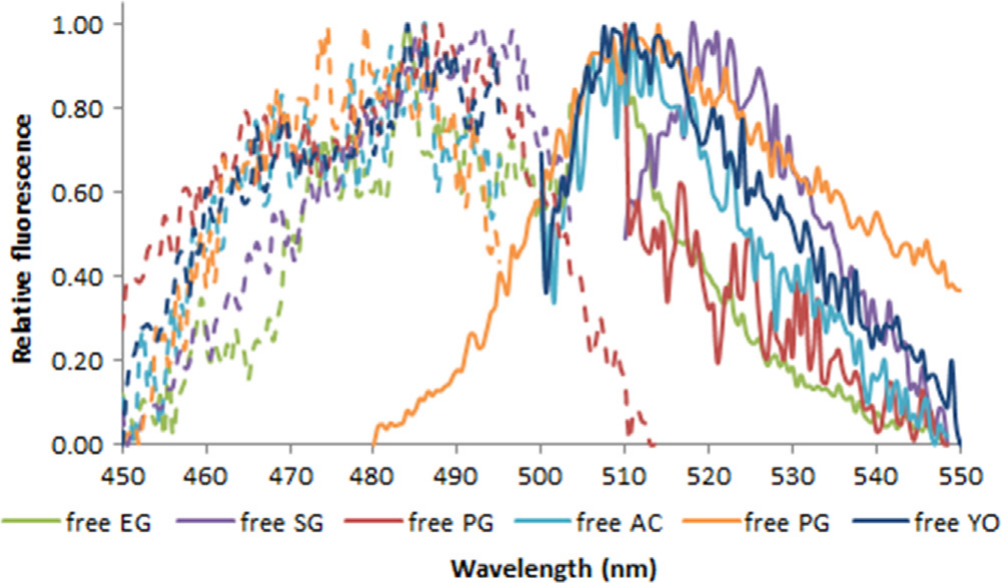
Excitation (dash lines) and emission (solid lines) spectra of 1.0X EvaGreen (green lines), SYBR Green (purple lines), PicoGreen (red lines), AccuClear (aqua lines), AccuBlue NextGen (orange lines) and 100 nM YOYO-1 (blue lines) free dye. Spectra were recorded with a LS55 fluorescence spectrometer (Perkin Elmer) (excitation slit: 2.5 nm, emission slit: 3.5 nm).

**Fig. 2.9:**
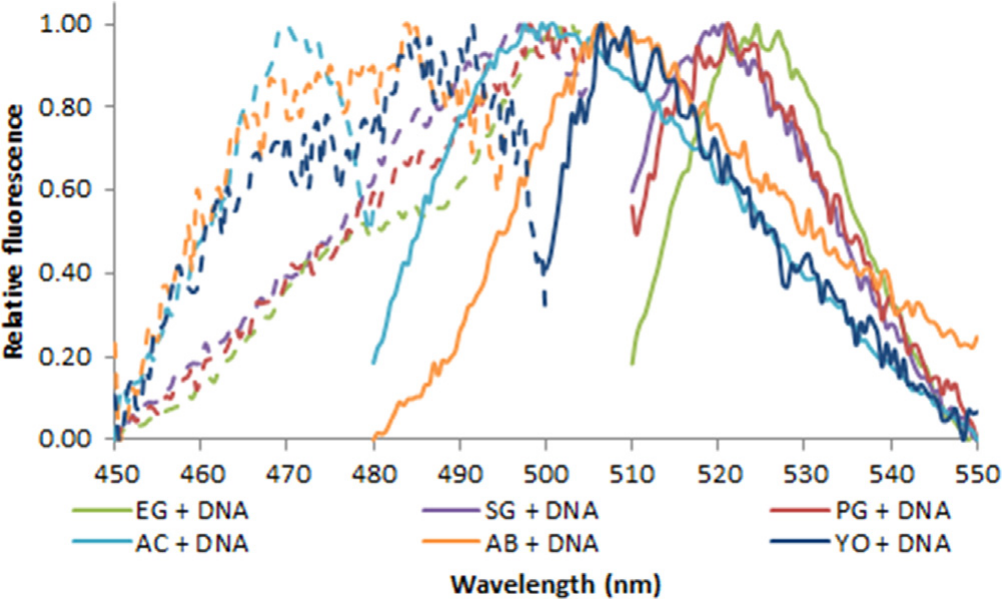
Excitation (dash lines) and emission (solid lines) spectra of 1.0X EvaGreen (green lines), SYBR Green (purple lines), PicoGreen (red lines), AccuClear (aqua lines), AccuBlue NextGen (orange lines) and 100 nM YOYO-1 (blue lines) in the presence of 50 ng/µL (4.76 ng/µL for AccuClear and AccuBlue NextGen) salmon dsDNA. Spectra were recorded with a LS55 fluorescence spectrometer (Perkin Elmer) (excitation slit: 2.5 nm, emission slit: 3.5 nm).

**Fig. 2.10:**
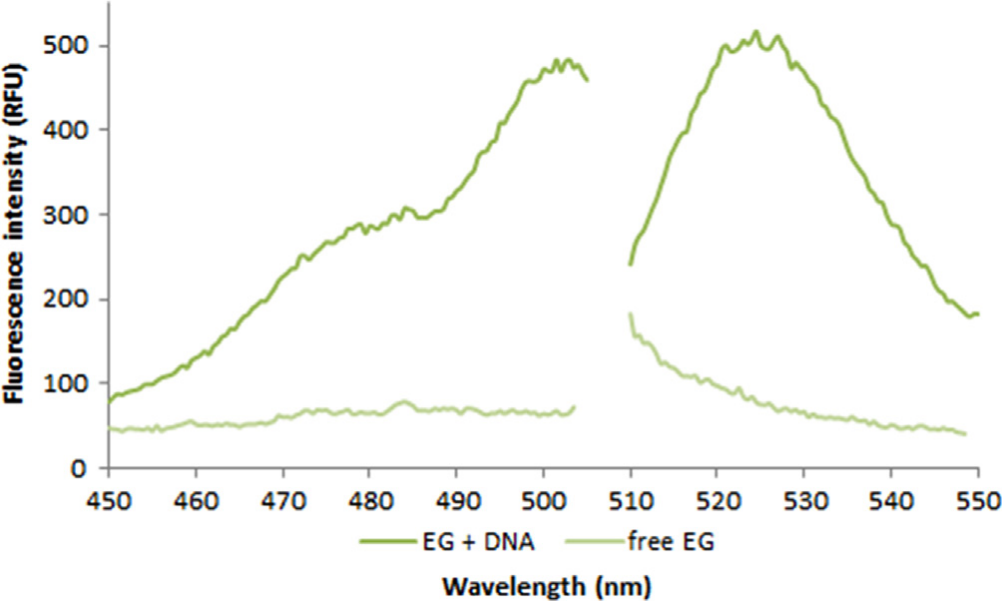
Excitation and emission spectra of 1.0X EvaGreen free dye (light green) and in the presence of 50 ng/µL salmon dsDNA (dark green). Spectra were recorded with a LS55 fluorescence spectrometer (Perkin Elmer) (excitation slit: 2.5 nm, emission slit: 3.5 nm).

**Fig. 2.11:**
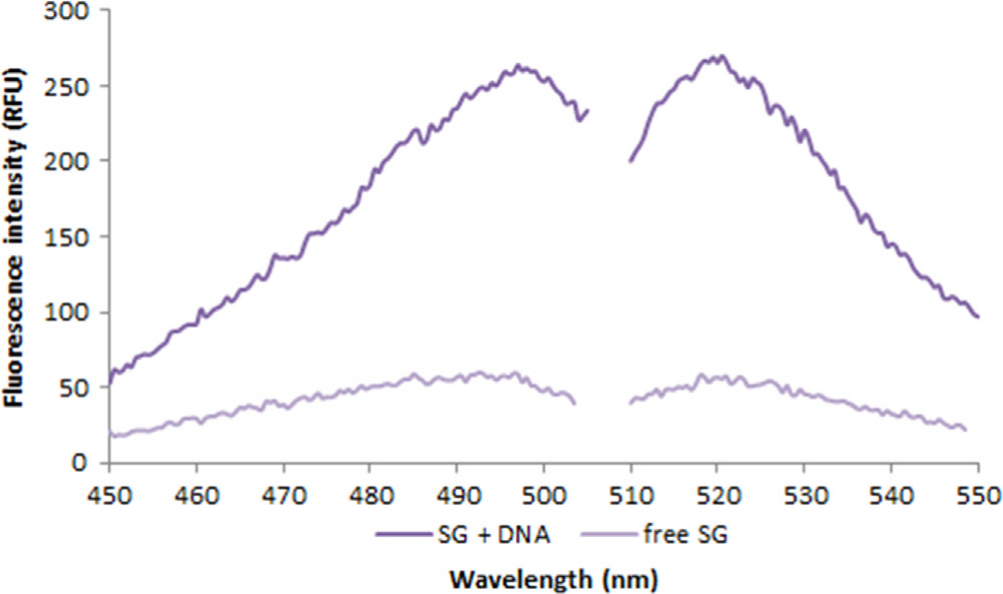
Excitation and emission spectra of 1.0X SYBR Green free dye (light purple) and in the presence of 50 ng/µL salmon dsDNA (dark purple). Spectra were recorded with a LS55 fluorescence spectrometer (Perkin Elmer) (excitation slit: 2.5 nm, emission slit: 3.5 nm).

**Fig. 2.12:**
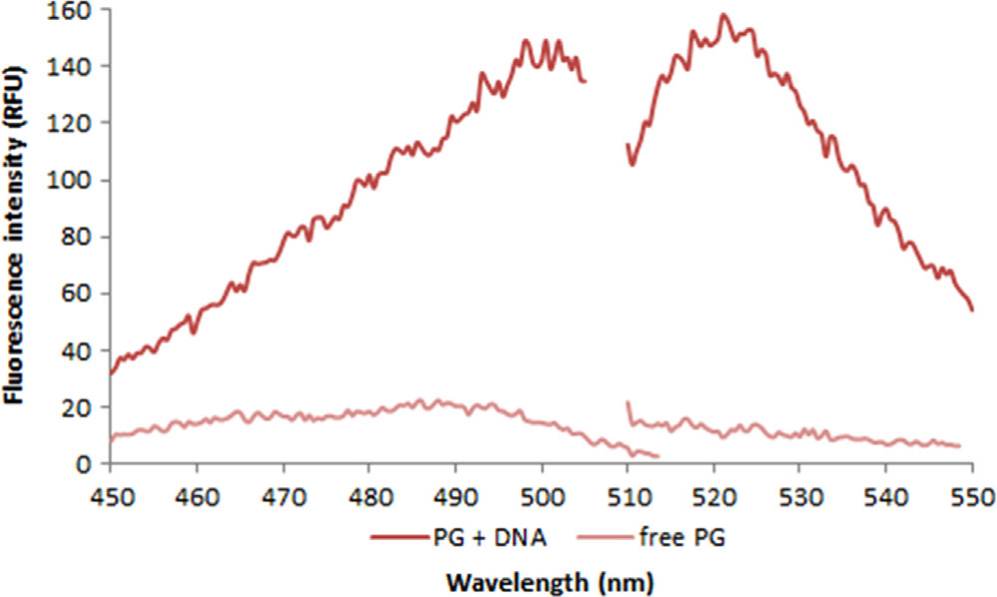
Excitation and emission spectra of 1.0X PicoGreen free dye (light red) and in the presence of 50 ng/µL salmon dsDNA (dark red). Spectra were recorded with a LS55 fluorescence spectrometer (Perkin Elmer) (excitation slit: 2.5 nm, emission slit: 3.5 nm).

**Fig. 2.13:**
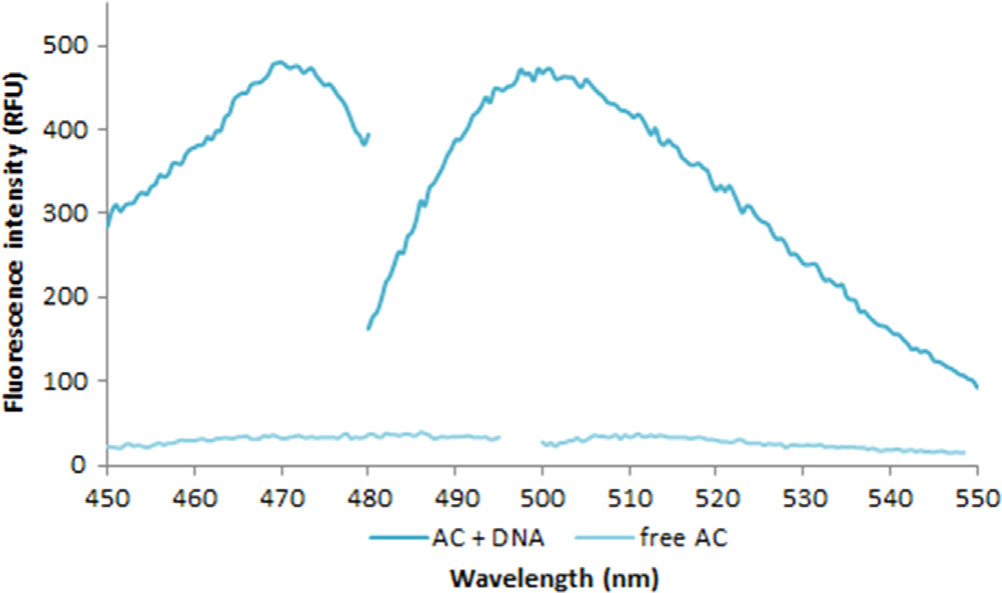
Excitation and emission spectra of 1.0X AccuClear free dye (light aqua) and in the presence of 4.76 ng/µL salmon dsDNA (dark aqua). Spectra were recorded with a LS55 fluorescence spectrometer (Perkin Elmer) (excitation slit: 2.5 nm, emission slit: 3.5 nm).

**Fig. 2.14:**
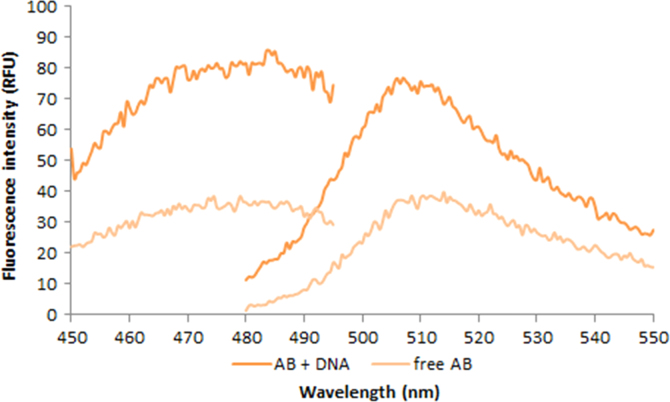
Left: Excitation and emission spectra of 1.0X AccuBlue NextGen free dye (light orange) and in the presence of 4.76 ng/µL salmon dsDNA (dark orange). Spectra were recorded with a LS55 fluorescence spectrometer (Perkin Elmer) (excitation slit: 2.5 nm, emission slit: 3.5 nm).

**Fig. 2.15:**
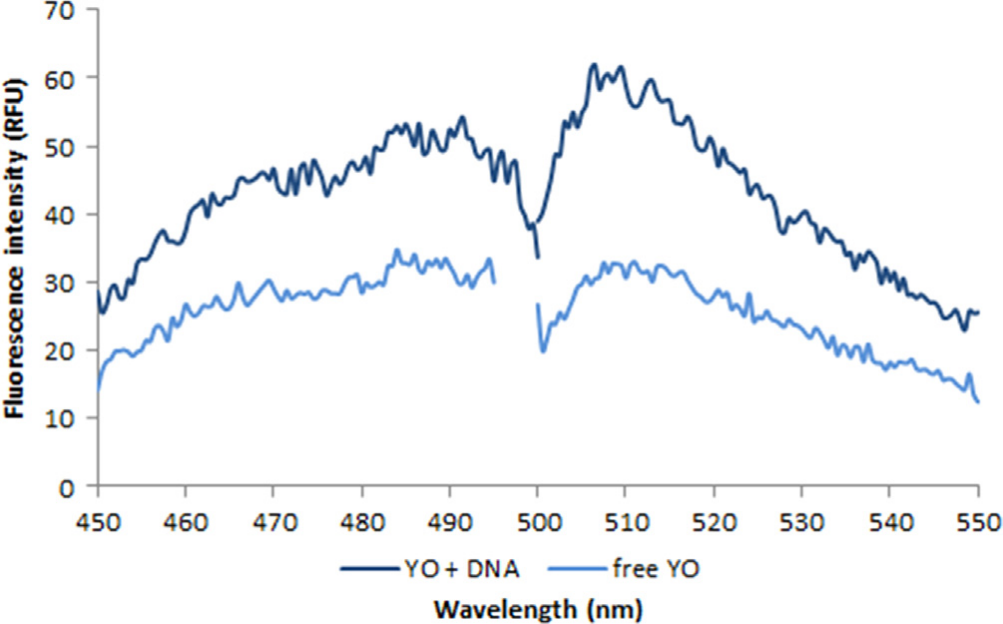
Left: Excitation and emission spectra of 100 nM YOYO-1 free dye (light blue) and in the presence of 50 ng/µL salmon dsDNA (dark blue). Spectra were recorded with a LS55 fluorescence spectrometer (Perkin Elmer) (excitation slit: 2.5 nm, emission slit: 3.5 nm).

**Fig. 2.16:**
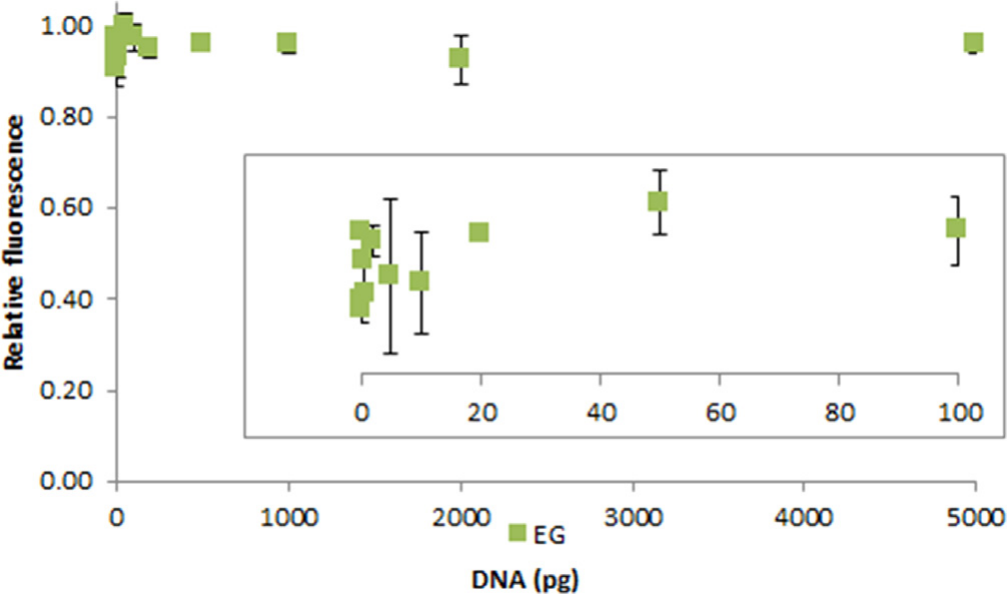
Relative fluorescence of 1.0X EvaGreen in the presence of 0–5000 pg salmon dsDNA. The insert shows the lower region of the curve. Spectra were recorded with a M200 PRO microplate reader (Tecan), gain 100. The error bars are ±1 standard deviation.

**Fig. 2.17:**
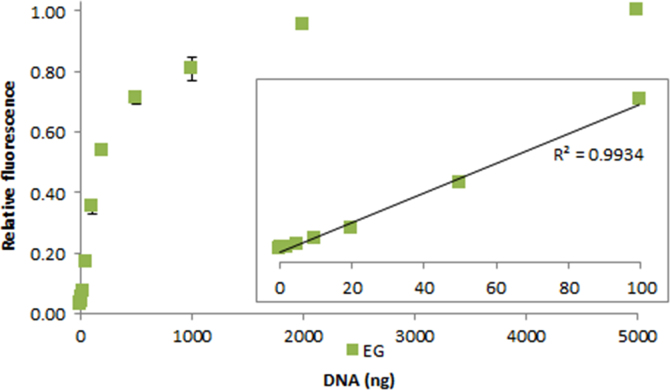
Relative fluorescence of 1.0X EvaGreen in the presence of 0–5000 ng salmon dsDNA. The insert shows the lower region of the curve with the *R*^2^-value given for 0.2–100 ng dsDNA. Spectra were recorded with a M200 PRO microplate reader (Tecan), gain 100. The error bars are ±1 standard deviation.

**Fig. 2.18:**
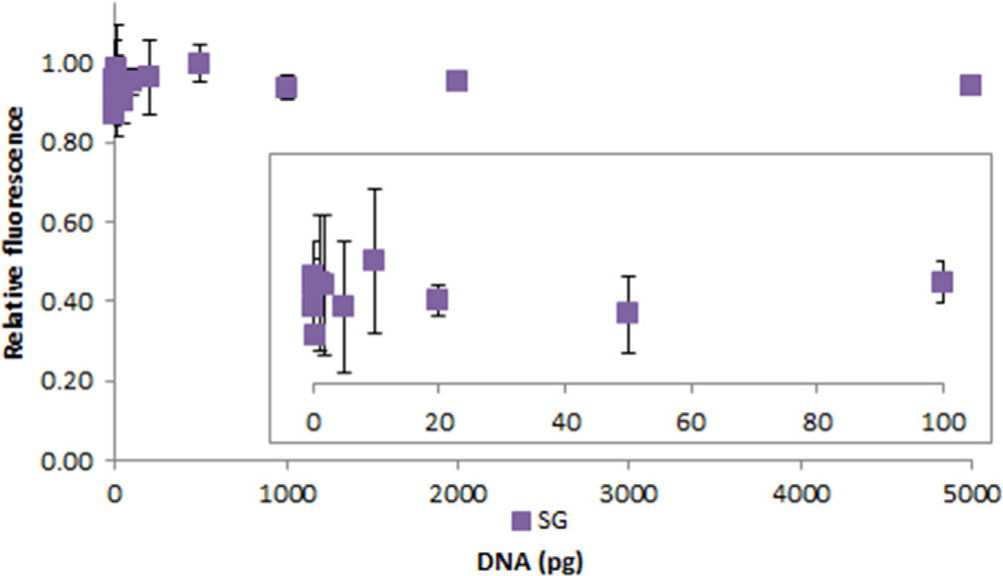
Relative fluorescence of 1.0X SYBR Green in the presence of 0–5000 pg salmon dsDNA. The insert shows the lower region of the curve. Spectra were recorded with a M200 PRO microplate reader (Tecan), gain 50. The error bars are ±1 standard deviation.

**Fig. 2.19:**
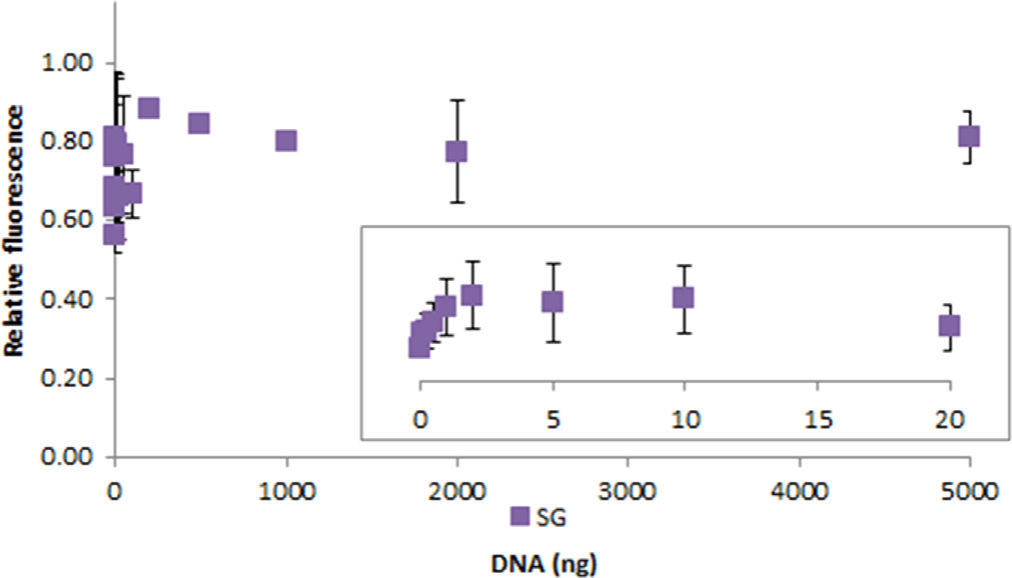
Relative fluorescence of 1.0X SYBR Green in the presence of 0–10,000 ng salmon dsDNA. The insert shows the lower region of the curve. Spectra were recorded with a M200 PRO microplate reader (Tecan), gain 50. The error bars are ±1 standard deviation.

**Fig. 2.20:**
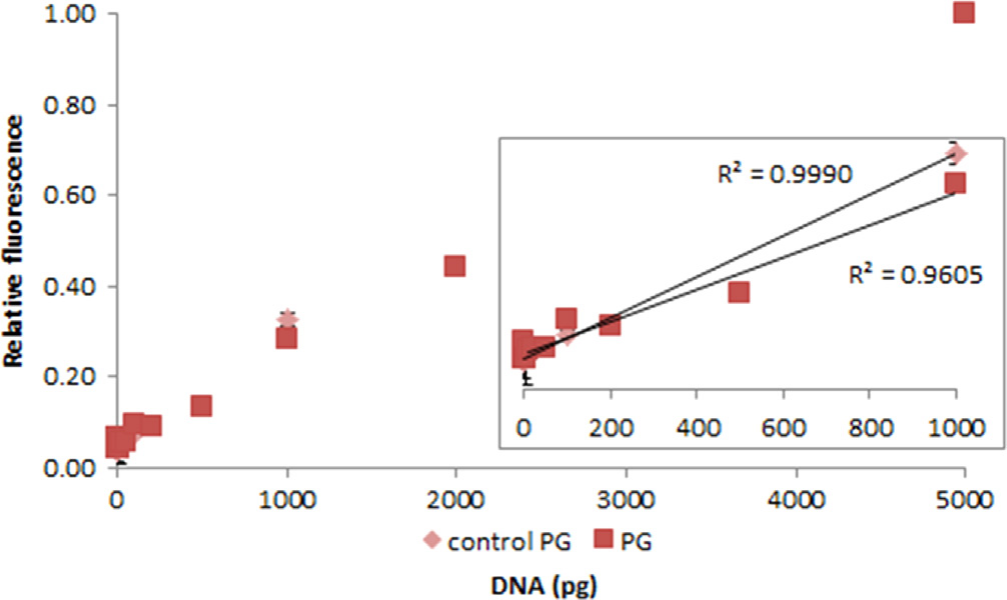
Relative fluorescence of 1.0X PicoGreen in the presence of 0–1000 pg control (light red diamonds) and 0–5000 pg salmon dsDNA (dark red squares). The insert shows the lower region of the curve, with the *R*^2^-value given for 10–1000 pg dsDNA. Spectra were recorded with a M200 PRO microplate reader (Tecan), gain 100. The error bars are ±1 standard deviation.

**Fig. 2.21:**
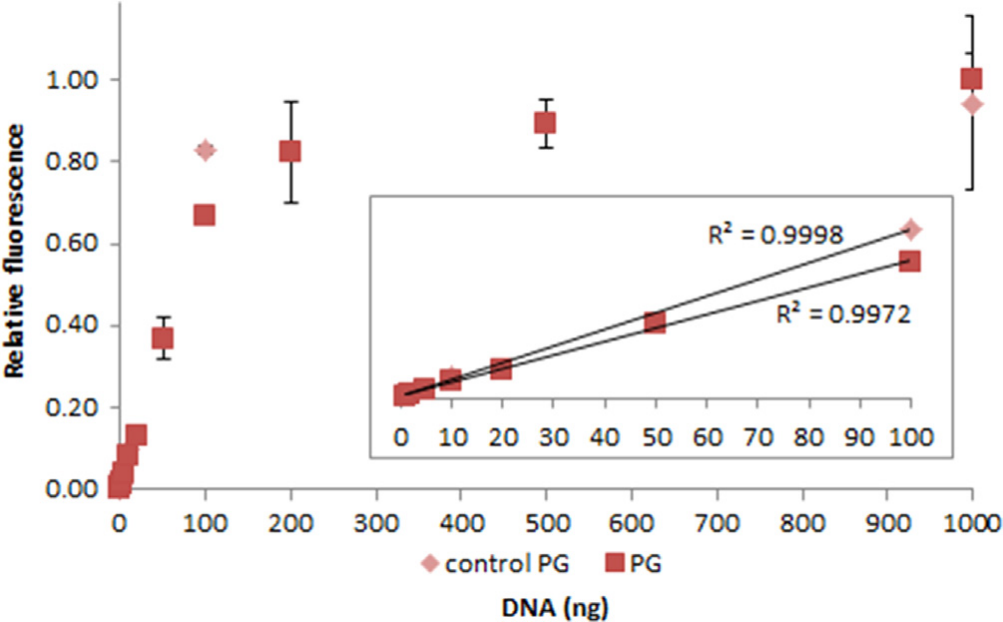
Relative fluorescence of 1.0X PicoGreen in the presence of 0–1000 ng control (light red squares) and salmon dsDNA (dark red diamonds). The insert shows the lower region of the curve, with the *R*^2^-value given for 1–100 ng dsDNA. Spectra were recorded with a M200 PRO microplate reader (Tecan), gain 100. The error bars are ±1 standard deviation.

**Fig. 2.22:**
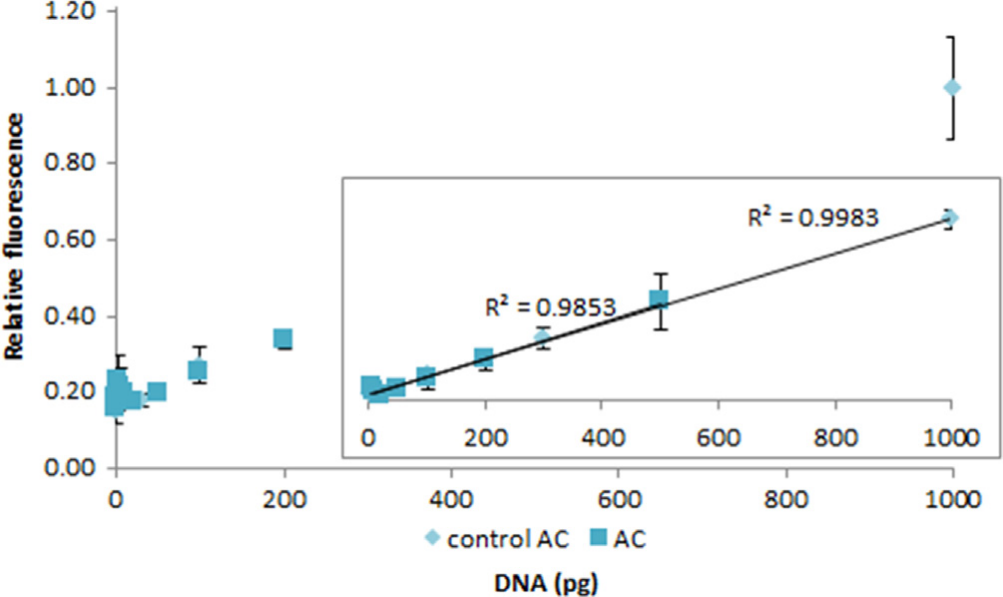
Relative fluorescence of 1.0X AccuClear in the presence of 0–1000 pg control (light aqua diamonds) and 0–500 pg salmon dsDNA (dark aqua squares). The insert shows the lower region of the curve, with the *R*^2^-value given for 3–1000 pg control dsDNA and 5–500 pg salmon dsDNA. Spectra were recorded with a M200 PRO microplate reader (Tecan), gain 100. The error bars are ±1 standard deviation.

**Fig. 2.23:**
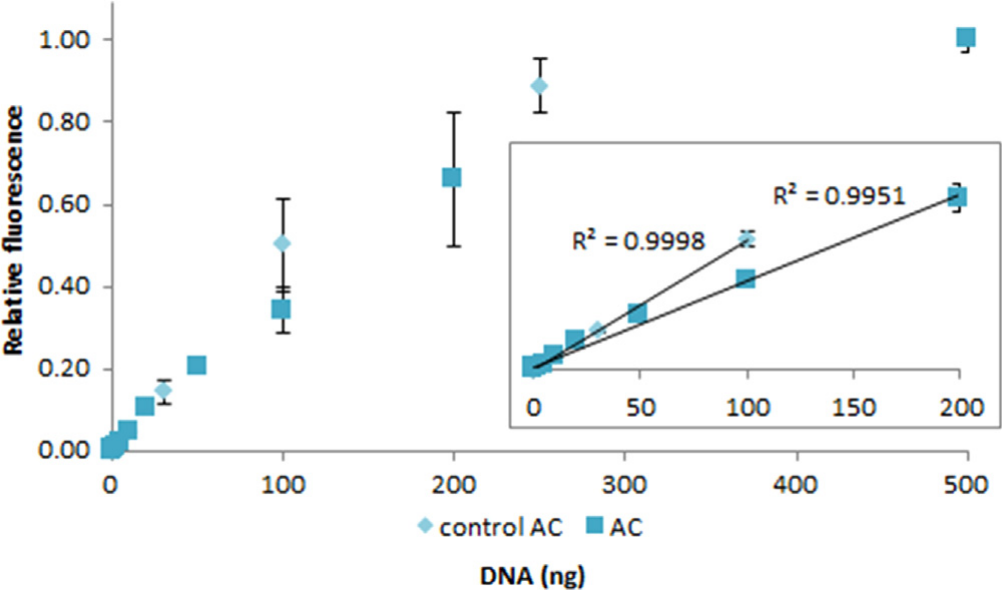
Relative fluorescence of 1.0X AccuClear in the presence of 0–250 ng control (light aqua diamonds) and 0–500 ng salmon dsDNA (dark aqua squares). The insert shows the lower region of the curve, with the *R*^2^-value given for 0.003–100 ng control dsDNA and 0.01–200 ng salmon dsDNA. Spectra were recorded with a M200 PRO microplate reader (Tecan), gain 100. The error bars are ±1 standard deviation.

**Fig. 2.24:**
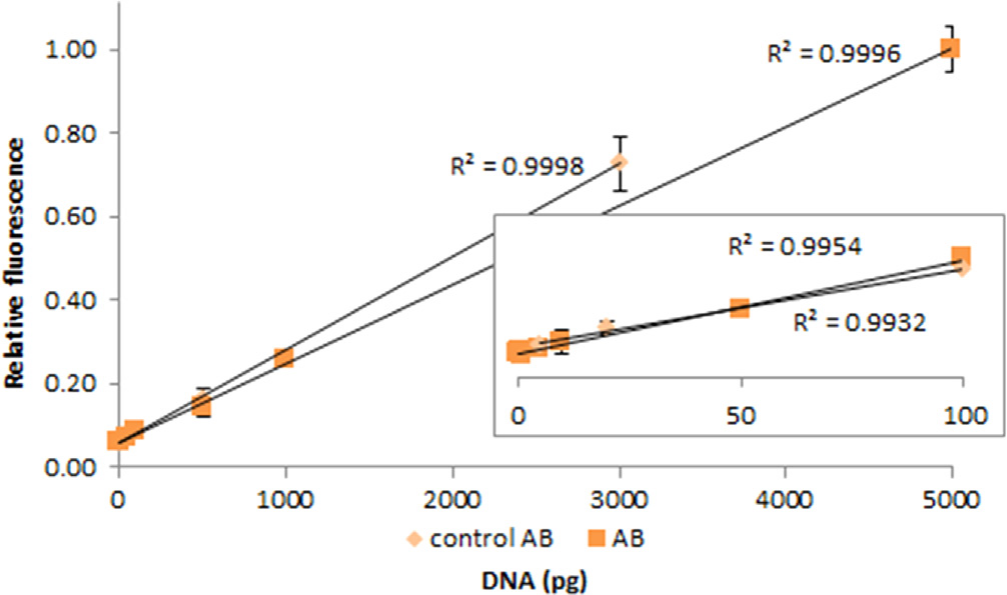
Relative fluorescence of 1.0X AccuClear in the presence of 0–3000 pg control (light orange diamonds) and 0–5000 pg salmon dsDNA (dark orange squares), with the *R*^2^-value. The insert shows the lower region of the curve, with the *R*^2^-value given for 5–100 pg control dsDNA and 0.1–100 pg salmon dsDNA. Spectra were recorded with a M200 PRO microplate reader (Tecan), gain 100. The error bars are ±1 standard deviation.

**Fig. 2.25:**
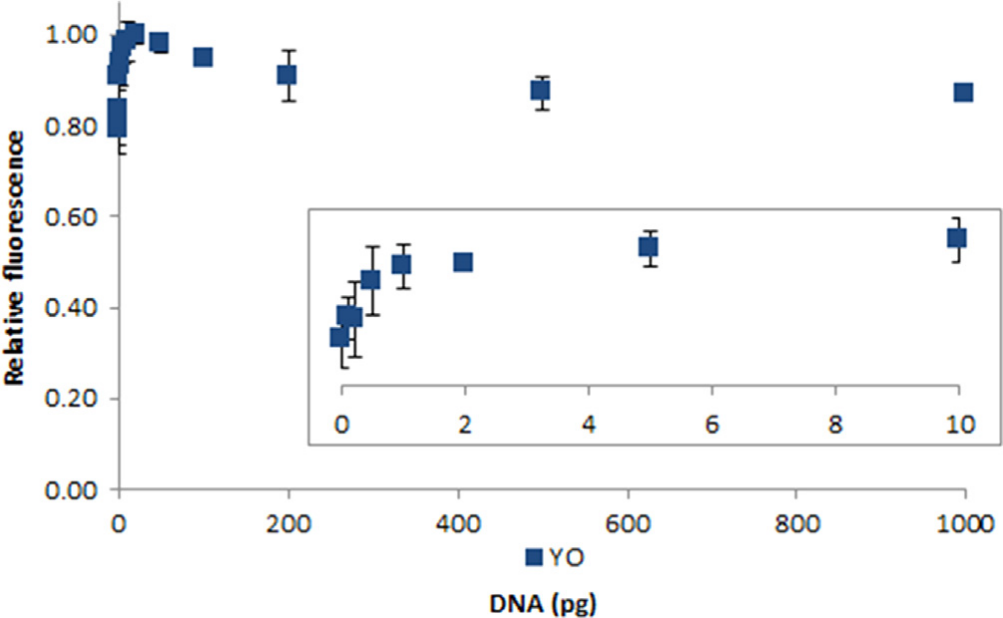
Relative fluorescence of 100 nM YOYO-1 in the presence of 0–1000 pg salmon dsDNA. The insert shows the lower region of the curve. Spectra were recorded with a M200 PRO microplate reader (Tecan), gain 50. The error bars are ±1 standard deviation.

**Fig. 2.26:**
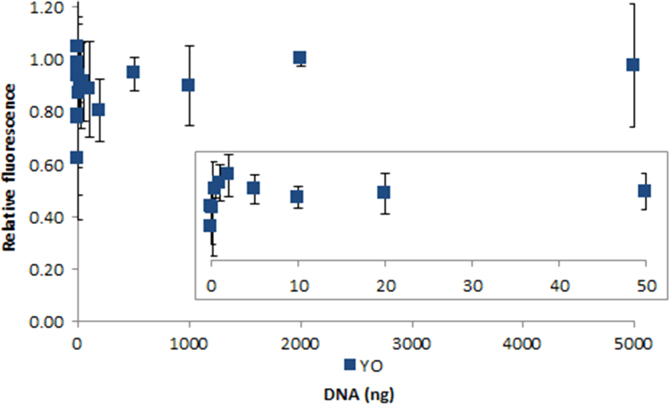
Relative fluorescence of 100 nM YOYO-1 in the presence of 0–5000 ng salmon dsDNA. The insert shows the lower region of the curve. Spectra were recorded with a M200 PRO microplate reader (Tecan), gain 50. The error bars are ±1 standard deviation.

**Table 1.1:** Absorption, excitation and emission wavelengths of various dyes free in solution and dye/dsDNA complexes.

Dye	Absorption		Excitation		Emission		Ref.
	Free	Complex	Free	Complex	Free	Complex	
EG				503 nm		527 nm	[2]
	470 nm	500 nm	495 nm	500 nm	525 nm	530 nm	[3]
				500 nm		529 nm	[4]
							
SG	494 nm	496 nm			530 nm	522 nm	[5]
	494 nm					524 nm	[6]
				497 nm		520 nm	[7]^a^
							
PG	498 nm	501 nm			528 nm	522 nm	[5]
				500 nm		523 nm	[8]
				480 nm		520 nm	[9]^a^[10]
				502 nm		523 nm	[9]^a^
		500 nm		480 nm		520 nm	[11]
							
AC		468 nm		468 nm		507 nm	[12]^a^
AB		468 nm		468 nm		507 nm	[13]^a^
							
YO	475 nm	490 nm			549 nm	507 nm	[5]
		491 nm		491 nm		509 nm	[14], [15]^a^
						
	460 nm	490 nm			560 nm	510 nm	[16]
	455 nm	485 nm				509 nm	[17]

a =information from the manufacturer of the dye.

## Footnotes

1 Since fluorescence is measured in relative fluorescence units (RFU), data cannot be directly compared. Therefore, relative intensities are used in most procedures; the data in most of the graphs are normalized. For the absorbance graphs with all the dyes also the background is subtracted (i.e. the lowest value equals 0).

Appendix A Supplementary data associated with this article can be found in the online version at http://dx.doi.org/10.1016/j.dib.2016.11.090.
