# Supplementary material for: Dataset of the absorption, emission and excitation spectra and fluorescence intensity graphs of fluorescent cyanine dyes for the quantification of low amounts of dsDNA
Source: Data Brief. 2016 Nov 28;10:132–43. doi: 10.1016/j.dib.2016.11.090 (PMC5143369; doi:10.1016/j.dib.2016.11.090)
Supplement: Supplementary file 1 — Supplementary material [file mmc1.docx]

Conflicts of interest: none

We wish to confirm that there are no known conflicts of interest associated with this publication and there has been no significant financial support fort his work that could have influenced its outcome.

On behalf of all co-authors,

Brigitte Bruijns
