# Supplementary material for: Dataset of the absorption, emission and excitation spectra and fluorescence intensity graphs of fluorescent cyanine dyes for the quantification of low amounts of dsDNA
Source: Data Brief. 2016 Nov 28;10:132–43. doi: 10.1016/j.dib.2016.11.090 (PMC5143369; doi:10.1016/j.dib.2016.11.090)
Supplement: Supplementary material [file mmc2.docx]

**Appendix A. Supplementary material**

Cyanine dyes are characterized by their absorption, excitation and emission spectra. The linearity of fluorescence, as function of DNA amount of these dyes, is obtained by measuring the fluorescence intensity at the optimal excitation and emission maxima. A literature based overview is given about the characteristics and spectral behaviour of the six researched dyes, such as the absorption, excitation and emission wavelengths at which maxima occur.

A shift in the absorption spectrum to higher wavelengths (bathochromic shift, informally referred to as a redshift) can be observed from the free dye in solution in comparison with the dye/dsDNA complex. The polarity in the dye's environment will decrease when a complex is formed, resulting in this shift. For the emission maxima a hypsochromic shift (blueshift) can be observed [1]. Upon intercalation of a bis-intercalating dsDNA dye (e.g. YOYO-1) between the bases of the DNA, the DNA molecule becomes distorted and the helix will unwind [2].

Note that for most dyes the absorption and excitation spectra are identical; for single fluorophores in homogeneous solutions this is usually true, but not in more complex heterogeneous solutions (if there are more molecules in the ground state (S0)). An absorption spectrum gives information at which wavelengths a molecule absorbs light and shows the S0-Sn (with n=1,2,...) bands. When a fluorophore is excited to a higher state it often ends up in the lowest excited state (S1) and emits radiation. In this case the excitation spectrum is the same as the absorption spectrum. The wavelength(s) necessary to obtain fluorescence can be determined by an excitation spectrum and only shows the S0-S1 band (and eventually S0-S2 and higher absorption bands, since after the excitation to S2 state the molecule quickly transits to the S1 state and after that fluorescence takes place) [2] [3].

The molecular structures of SYBR Green, PicoGreen and YOYO-1 are depicted in Figure 1.1 [4, 5, 6]. In this figure also a suggestion for EvaGreen is given. Although the exact structure of EvaGreen is not given by the manufacturer, it is assumed that this molecule is formed by two homo-monomers linked by a bridge. The molecular structures of AccuClear and AccuBlue NextGen are not disclosed by the manufacturer.


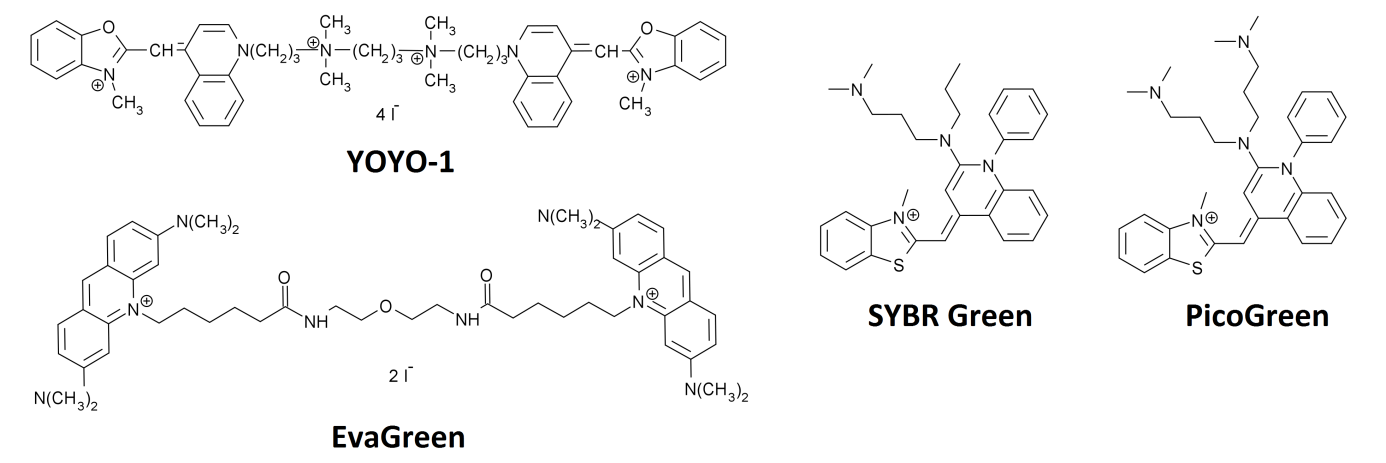


Figure 1.1. Molecular structures of YOYO-1, EvaGreen, SYBR Green and PicoGreen [4, 5, 6].

**EvaGreen**

EvaGreen is designed for real-time quantitative PCR applications [7]. This dimeric bis-intercalating dye shifts between an inactive looped conformation that does not bind DNA at low DNA concentrations, for example at the beginning of a real-time PCR reaction, and an active conformation, which is stable at higher DNA concentrations [8] [9]. It is believed that the inactive looped conformation, also called hairpin structure, is minimally fluorescent by itself, but in the presence of nucleic acids becomes highly fluorescent [10].

Pure EvaGreen, with a concentration of 11.15 µM, shows absorption at 470 nm (major peak) and at 495 nm (shoulder peak) according to Mao et al. For increasing amounts of λDNA, from 0 ng/µL up to 100 ng/µL, the shoulder peak disappears and a peak emerges at 500 nm, which becomes the major peak at higher DNA concentrations. Mao et al. claimed that at saturation conditions (40 ng/µL DNA and 11.42 µM EvaGreen) the enhancement in fluorescence is about 70 times [8]. Ihrig et al. found saturation conditions for 1.0X (1.33 µM) EvaGreen at 500 ng in 100 µL [7]. In both cases this corresponds to a ratio of 1 EvaGreen molecule per 5 to 6 basepairs.

Wang et al. concluded that the fluorescence intensity of EvaGreen is proportional to the total amount of DNA present and not necessarily to the concentration of the DNA present. They showed a linear response of the relative fluorescence in the range of 0-100 ng of DNA for 1.0X EvaGreen. However, DNA quantification less than 1 ng is not recommended, due to the fact that the linearity in that region was not reliable [11].

**SYBR Green**

SYBR Green is a sensitive nucleic acid gel stain for the detection of dsDNA in agarose and polyacrylamide gels [12]. SYBR Green, determined as [2-(N-(3-dimethylaminopropyl)-N-propylamino}-4-(2,3-dihydro-3-methyl-(benzo-1,3-thiazol-2-yl)-methylidene)-1-phenyl-quinolinium], most likely is a minor groove binding dye (at high dye:base pair ratios [4]. The dye has a minor preference for AT-rich sequences, mainly at higher dye:base pair ratios [4] [8].

The excitation maximum of SYBR Green lies at 497 nm, but also excitation at 290 nm and 380 nm is possible (secondary excitation peaks) [12].

Zipper et al. discovered that the fluorescence intensity of SYBR Green bound to dsDNA can be divided in three stages. At a low dye:base pair ratio the intensity increases only slightly (up to about a ratio of 0.15). At higher ratios, 0.15-2, an abrupt increase in fluorescence intensity was observed. Above 2.5 a lower increase was measured up to a maximum intensity at a ratio of 10 [4].

**PicoGreen**

The structure of PicoGreen is known and the systematic name of the molecule is [2-(N-bis-(3-dimethylaminopropyl)-amino)-4-(2,3-dihydro-3-methyl-(benzo-1,3-thiazol-2-yl)-methylidene)-1-phenyl-quinolinium]. Dragan et al. suggested that binding of PicoGreen to DNA takes place by the positively charged benzo-thiazol group of PicoGreen, which is electrostatically bound to the DNA phosphate group, but can also form extensive contacts within the DNA groove(s). The quinolinium group intercalates into the DNA and is further stabilized by Van der Waals interactions [13]. Therefore this dye has no preferences for specific sequences in the DNA [13] [14].

Although the recommended wavelengths to use for the complex for excitation and emission are 480 nm and 520 nm, respectively [13] [15] [16], the excitation and emission maxima are measured at 502 nm and 523 nm, respectively [1] [14] [15].

**AccuClear & AccuBlue NextGen**

AccuClear, as well as AccuBlue NextGen, are new intercalating dsDNA quantitation dyes from Biotium. AccuBlue is available as a broad range kit (linear range: 2-2000 ng dsDNA) and as a high sensitivity kit (linear range: 0.2-200 ng). For ultra high sensitivity the AccuClear kit is recommended with a linear range of 0.03-250 ng dsDNA. This dye is green fluorescent with an excitation (and absorption) and emission maximum of 468 nm and 507 nm, respectively. Recently the AccuBlue NextGen kit has been developed. A linear range of 1-3000 pg has been claimed (lowest amount of DNA detected depends on the used reader). This kit has the same spectral properties as the AccuClear dye. No information is given on the molecular structures of these dyes [17] [18].

**YOYO-1**

YOYO-1, [1,1-(4,4,7,7-tetramethyl-4,7-diazaunde-camethylene)-bis-4-(3-methyl-2,3-dihydro-(benzo-1,3-oxazole)-2-methylidene)-quinolinium tetraiodide)], is an oxacyanine dye with one carbon atom bridging the aromatic rings [2] [19]. YOYO-1 reveals at least two binding modes; one for low dye:base pair ratios and one for high dye:base pair ratios. In the first case the dye behaves as a bis-intercalator, whereby each monomer unit intercalates (the benzazolium ring between the pyrimidines and the quinolium ring between the purine rings). External binding, a less well characterized mode, begins to contribute at higher ratios [5].

Absorption spectra of YOYO-1, free dye in aqueous buffered solutions, show maxima around 460 nm (major peak) and at 485 nm (shoulder peak). When bound to DNA a maximum at 490 nm and a shoulder peak at 465 nm appear for increasing amounts of double-stranded salmon sperm DNA [5].

The highest fluorescence intensity is obtained with a DNA base pair:dye ratio of 5:1 (compared to ratios of 10:1, 20:1 and 30:1 [19]. For high dsDNA concentrations (2.1 µg/mL) the fluorescence intensity decreased when the dye:base pair ratio approached 1:1. With low dsDNA concentrations (95 ng/mL) the intensity reached a plateau level with ratios of 1-2 dye molecules per base pair. Rye et al. assume that the loss of fluorescence at high dsDNA concentrations is the result of dye-induced aggregation of the DNA. Besides this, the binding of the dye, which shows non-intercalative modes at high dye:base pair ratios, could lead to quenching of the intercalated dye molecules [20].

**References**

| [1] | G. Cosa, K. Focsaneanu, J. McLean, J. McNamee and J. Scaiano, “Photophysical properties of fluorescent DNA-dyes bound to single-and double-stranded DNA in aqueous buffered solution,” *Photochemistry and Photobiology,* vol. 73, no. 6, pp. 585-599, 2001. |
| --- | --- |
| [2] | Thermo Fisher Scientific, The Molecular Probes Handbook; A guide to fluorescent probes and labeling technologies, Life Technologies Corporation, 2010. |
| [3] | D. Skoog, F. Holler en S. Crouch, Principles of Instrumental Analysis, Belmont: Thomson Higher Education, 2007. |
| [4] | H. Zipper, H. Brunner, J. Bernhagen and F. Vitzthum, “Investigations on DNA intercalation and surface binding by SYBR Green I, its structure determination and methodological implications,” 2004. [Online]. Available: doi: 10.1093/nar/gnh101. |
| [5] | A. Fürstenberg, M. Julliard, T. Deligeorgiev, N. Gadjev, A. Vasilev and E. Vauthey, “Ultrafast excited-state dynamics of DNA fluorescent intercalators: new insight into the fluorescence enhancement mechanism,” 2006. [Online]. Available: doi:10.1021/ja0609001. |
| [6] | I. Kavanagh, D. Leake en G. Ball, „Dye blends”. Patent Patent US20130052650 A1, 28 February 2013. |
| [7] | J. Ihrig, R. Lill and U. Mühlenhoff, “Application of the DNA-specific dye EvaGreen for the routine quantification of DNA in microplates,” 2006. [Online]. Available: doi:10.1016/j.ab.2006.07.043. |
| [8] | F. Mao, W. Leung and X. Xin, “Characterization of EvaGreen and the implication of its physicochemical properties for qPCR applications,” 2007. [Online]. Available: doi:10.1186/1472-6750-7-76. |
| [9] | Biotium, „EvaGreen Dye, 20X in water,” 2015. [Online]. Available: https://biotium.com/product/evagreen-dye-20x-in-water. |
| [10] | F. Mao, W.-Y. Leung and T. Van, “Methods of using dyes in association with nucleic acid staining or detection”. Patent US Patent 8,877,437, 2014. |
| [11] | W. Wang, K. Chen and C. Xu, “DNA quantification using EvaGreen and a real-time PCR instrument,” 2006. [Online]. Available: doi:10.1016/j.ab.2006.05.027. |
| [12] | Thermo Fisher Scientific, “SYBR Green I Nucleic Acid Gel Stain, 10,000X concentrate in DMSO,” 2015. [Online]. Available: https://www.lifetechnologies.com/order/catalog/product/S7567. |
| [13] | A. Dragan, J. Casas-Finet, E. Bishop, R. Strouse, M. Schenerman and C. Geddes, “Characterization of PicoGreen interaction with dsDNA and the origin of its fluorescence enhancement upon binding,” 2010. [Online]. Available: doi:10.1016/j.bpj.2010.09.012. |
| [14] | V. Singer, L. Jones, S. Yue and R. Haugland, “Characterization of PicoGreen reagent and development of a fluorescence-based solution assay for double-stranded DNA quantitation,” 1997. [Online]. Available: doi:10.1006/abio.1997.2177. |
| [15] | Thermo Fisher Scientific, “Quant-iT PicoGreen dsDNA Assay Kit,” 2015. [Online]. Available: https://www.lifetechnologies.com/order/catalog/product/P7589. |
| [16] | G. Schofield, “PicoGmeter, a custom-made fluorometer for the quantification of dsDNA by PicoGreen fluorescence,” *BioTechniques,* vol. 37, no. 5, pp. 778-782, 2004. |
| [17] | Biotium, “dsDNA quantitation kits; AccuBlue and AccuClear dsDNA Quantitation Kits for fluorescence-based dsDNA quantitation in solution,” 2015. [Online]. Available: https://biotium.com/product-category/applications/genomics/nucleic-acid-quantitation-in-solution. |
| [18] | Biotium, “AccuBlue NextGen dsDNA Quantitation Kit,” 2015. [Online]. Available: https://biotium.com/product/accublue-nextgen-dsdna-quantitation-kit. |
| [19] | H. Rye, S. Yue, D. Wemmer, M. Quesada, R. Haugland, R. Mathies and A. Glazer, “Stable fluorescent complexes of double-stranded DNA with bis-intercalating asymmetric cyanine dyes: properties and applications,” *Nucleic Acids Research,* vol. 20, no. 11, pp. 2803-2812, 1992. |
| [20] | H. Rye, J. Dabora, M. Quesada, R. Mathies and A. Glazer, “Fluorometric assay using dimeric dyes for double-and single-stranded DNA and RNA with picogram sensitivity,” 1993. [Online]. Available: doi: 10.1006/abio.1993.1020. |
